# Antibiotic exposure and acquisition of antibiotic-resistant gram-negative bacteria among outpatients at a US Veterans Affairs medical center

**DOI:** 10.1017/ash.2021.231

**Published:** 2022-01-12

**Authors:** Ukwen C. Akpoji, Brigid M. Wilson, Janet M. Briggs, Sunah Song, Taissa A. Bej, Federico Perez, Robin L. P. Jump

**Affiliations:** 1 Department of Pharmacy, Veterans Affairs Northeast Ohio Healthcare System, Cleveland, Ohio; 2 Geriatric Research Education and Clinical Center (GRECC), VA Northeast Ohio Healthcare System, Cleveland, Ohio; 3 Division of Infectious Diseases & HIV Medicine in the Department of Medicine, Case Western Reserve University School of Medicine, Cleveland, Ohio; 4 Cleveland Institute for Computational Biology, Case Western Reserve University School of Medicine, Cleveland, Ohio; 5 Case Western Reserve University–Cleveland VAMC Center for Antimicrobial Resistance and Epidemiology (Case VA CARES), Cleveland, Ohio; 6 Department of Population and Quantitative Health Sciences, Case Western Reserve University School of Medicine, Cleveland, Ohio

## Abstract

**Objectives:**

To assess the prevalence of antibiotic-resistant gram-negative bacteria (R-GNB) among patients without recent hospitalization and to examine the influence of outpatient antibiotic exposure on the risk of acquiring R-GNB in this population.

**Design:**

2-year retrospective cohort study.

**Setting:**

Regional Veterans Affairs healthcare system.

**Patients:**

Outpatients at 13 community-based clinics.

**Methods:**

We examined the rate of acquisition of R-GNB within 90 days following an outpatient visit from 2018 to 2019. We used clinical and administrative databases to determine and summarize prescriptions for systemic antibiotics, associated infectious diagnoses, and subsequent R-GNB acquisition among patients without recent hospitalizations. We also calculated the odds ratio of R-GNB acquisition following antibiotic exposure.

**Results:**

During the 2-year study period, 7,215 patients had outpatient visits with microbiological cultures obtained within 90 days. Of these patients, 206 (2.9%) acquired an R-GNB. Among patients receiving antibiotics at the visit, 4.6% acquired a R-GNB compared to 2.7% among patients who did not receive antibiotics, yielding an unadjusted odds ratio of 1.75 (95% confidence interval, 1.18–2.52) for a R-GNB following an outpatient visit with versus without an antibiotic exposure. Regardless of R-GNB occurrence, >50% of antibiotic prescriptions were issued at visits without an infectious disease diagnosis or issued without documentation of an in-person or telehealth clinical encounter.

**Conclusions:**

Although the rate of R-GNBs was low (2.9%), the 1.75-fold increased odds of acquiring a R-GNB following an outpatient antibiotic highlights the importance of antimicrobial stewardship efforts in outpatient settings. Specific opportunities include reducing antibiotics prescribed without an infectious diagnosis or a clinical visit.

Most patients receive their health care in outpatient settings, culminating in nearly 1 billion office visits annually.^1^ From 2010 to 2011, ∼13% of outpatient visits resulted in an antibiotic prescription, of which an estimated 30% were likely inappropriate.^2^ Injudicious prescriptions of antibiotics in outpatient settings are associated with billion-dollar healthcare expenditures and contribute to the public health threat of antibiotic-resistant bacteria, including an array of gram-negative pathogens.^3,4^ Recent data indicate that an increasing proportion of hospitalizations for infections caused by antibiotic-resistant gram-negative bacteria (R-GNB), particularly extended-spectrum β-lactamase–producing Enterobacterales (ESBLs), are community associated.^5–8^ These reports indicate a pressing need to better understand risk factors associated with acquisition of R-GNB in community settings.

Hospital settings have made strides in reducing the risk of acquisition, transmission, and infection due to R-GNB through the advancement of antibiotic stewardship among inpatients as well as other infection control and prevention measures.^9–11^ Whether improved antibiotic stewardship in the outpatient setting can achieve similar effects has not been firmly established, in part because the contribution of outpatient antibiotic exposure to the risk of acquiring R-GNB is not well characterized.^12,13^ Unlike allergic reactions or other adverse effects that come to clinical attention within hours to days of antibiotic administration, events related to acquisition of antibiotic-resistant bacteria can occur weeks after an antibiotic exposure and, in the case of colonization, may never come to clinical attention. Care received across separate healthcare systems, including hospitals and other outpatient settings can further obscure efforts to quantify the relationship between any given antibiotic exposure and the subsequent acquisition of antibiotic-resistant bacteria.^2,14^

The Veterans Health Administration (VHA) uses a common electronic medical record to document and support comprehensive care for patients across the continuum of healthcare, which include hospitals, nursing homes, emergency departments, as well as clinics for primary, urgent, and specialty care. Furthermore, pharmacy and laboratory records, including antibiotic prescriptions and results of microbiological cultures, are also fully embedded within the same system. This integrated EMR facilitates assessment of a wide array of factors potentially associated with acquisition of R-GNB. To assess the risk factors related to outpatients acquiring R-GNB, we conducted a retrospective cohort study of veterans who received care at community-based outpatient clinics associated with a large Veterans Affairs medical center (VAMC). The objectives of our study were to assess the incidence of R-GNB among outpatients and to examine risk factors associated with acquisition of R-GNB. The exposure of interest was prescription of antibiotics in the outpatient setting.

## Methods

### Study design and data sources

We conducted a retrospective study of patients who received care at any of 13 free-standing community-based outpatient clinics associated with the VA Northeast Ohio Healthcare System (VA NEOHS) between January 1, 2018, and December 31, 2019. We used the VA Informatics and Computing Infrastructure (VINCI) to access the VHA Corporate Data Warehouse (CDW), which contains integrated databases from national clinical and administrative data sets, to identify the study population.^15^ The Institutional Review Board of the VA Northeast Ohio Healthcare System approved the study protocol.

### Study population

In this study, we included patients who had at least 1 outpatient encounter with a community-based primary care provider during the study period with a microbiological culture collected 7–90 days following that encounter. The microbiological cultures were used to assess the outcome of interest, acquisition of R-GNB. The cultures could have been collected in the outpatient setting or within 72 hours of a hospital admission because pathogens identified within this window are considered community acquired. We considered patients who received an antibiotic prescription issued through a primary care provider in the absence of a documented clinical encounter (ie, telephone prescribing that occurred >1 week removed from a documented outpatient encounter) to have had a “no clinic” outpatient encounter.

We excluded patients who were prescribed an antibiotic or were discharged from a hospital or nursing home in the 90 days before the qualifying outpatient encounter with their primary care provider. We also excluded patients who were prescribed a new antibiotic >7 days after their qualifying outpatient encounter but prior to their first microbiological culture, as this represented a change in their exposure status during the R-GNB follow-up period. Lastly, we excluded patients with a documented history of R-GNB prior to the qualifying outpatient visit. This was determined by identifying in the microbiological records of the VA healthcare system cultures from any specimen growing any of the following gram-negative bacteria: *Pseudomonas aeruginosa*, *Escherichia coli*, *Citrobacter koseri,* and *Klebsiella*, *Enterobacter*, *Serratia*, *Proteus*, and *Providencia* spp. Organisms with antimicrobial susceptibility results other than what is considered the wild type for the species were considered to represent a R-GNB. *P. aeruginosa* resistance was based on the following agents: fluoroquinolones, cefepime, piperacillin–tazobactam, and imipenem–cilastin. For the remaining GNB, we considered the following 4 phenotypes to indicate resistance: fluoroquinolone resistance; resistance to extended-spectrum cephalosporins (eg, ceftriaxone and cefepime); resistance to β-lactam–β-lactamase inhibitor combinations (eg, ampicillin-sulbactam and piperacillin-tazobactam), and resistance to carbapenems (Supplementary Tables 1A–E). Our definitions for R-GNB were consistent with recently published guidance.^16^ We conducted a manual chart review in a subset of 30 patients to validate the R-GNB definition applied to the electronic clinical and administrative databases utilized in this study.

**Table 1.** Characteristics of Patients with an Outpatient Visit Followed by Microbiological Cultures in 2018–2019

Patient Characteristics	No Resistant Gram-Negative Bacteria (n = 7,009)	Resistant Gram-Negative Bacteria (n = 206)	*P* Value^a^
Sex, male, no. (%)	6,355 (91)	172 (83)	.001
Age at encounter, mean y (± SD)	65.4 (±14.3)	68.1 (±13.4)	.006
**Race, no. (%)**			.011
White	5,868 (84)	160 (78)	
Black	842 (12)	39 (19)	
Other^b^	299 (4)	7 (3)	
**Ethnicity, no. (%)**			.923
Non-Hispanic	6,752 (96)	198 (96)	
Hispsanic	117 (2)	3 (1)	
Other^b^	140 (2)	5 (2)	
Charlson comorbidity index, mean (± SD)	1.88 (± 2.0)	1.88 (± 2.1)	.997
**Specific medical comorbid conditions, no. (%)**			
Diabetes mellitus	2,437 (35)	71 (34)	.987
Chronic lung disease	1,715 (24)	62 (30)	.077
Cancer	951 (14)	23 (11)	.373
Peripheral vascular disease	948 (14)	27 (13)	.944
Chronic renal disease	831 (12)	22 (11)	.685
Stroke	804 (11)	28 (14)	.407
Cardiac disease	686 (10)	28 (14)	.092
Liver disease	535 (8)	17 (8)	.844
**Healthcare events in the year prior to the index visit, no. (%)^c^**			
Outpatient antibiotic prescription (≥1)^d^	1,374 (20)	43 (21)	.716
Hospital admission (≥1)^d^	566 (8)	18 (9)	.831
Emergency department visits (≥1)	1,160 (17)	24 (12)	.076
Specialty care visits, median (± IQR)	5 (2–11)	4 (2–9)	.086
Primary care visits, median (± IQR)	3 (1–4)	2 (1–4)	.068
Antibiotic prescribed at index visit	711 (10)	34 (17)	.004

Note. SD, standard deviation; IQR, interquartile range.

a *P* values reflect independent samples t-tests for age and Charlson comorbidity index, Wilcoxon rank-sum tests for primary care and specialty care visit counts, and χ^2^ tests for categorical variables. All *P* values shown are unadjusted.

b For race, other includes American Indian, Alaska Native, Asian, Native Hawaiian or Pacific Islander, multiple and unknown. For ethnicity, other includes multiple and unknown.

c Limited to visits and prescriptions within the VA Northeast Ohio Healthcare System.

d Accounts for the year prior to the index visit; patients who were discharged or who received outpatient antibiotics ≤90 days prior to their index visit were excluded from analysis.

For each patient remaining in the cohort, we identified the first qualifying outpatient encounter as our index visit for analysis. Antibiotics prescribed at the index visit was our exposure of interest. Antibiotics prescribed in the same clinic within 7 days of the index visit were considered linked to the index visit.

The following data were obtained from the CDW and were used to describe the patients at their index visit: age, sex, race, ethnicity and, using *International Classification of Diseases, Ninth Revision* (ICD-9) and *Tenth Revision* (ICD-10) codes, and common infectious diseases diagnoses and comorbid conditions, from which the Charlson comorbidity index (CCI) was derived.^17^ We also assessed encounters in the VA NEOHS in the year prior to the index visit, including antibiotic exposures and the number of primary care visits, outpatient visits with a specialist, emergency room visits, and hospital admissions. Antibiotic prescriptions and infectious disease diagnoses were assessed for each patient’s index visit. Infectious disease diagnoses associated with visits were based on ICD-10 codes and classified into common infectious syndromes, as previously described.^18^

### Data analysis and statistical methods

The primary outcome was the acquisition of any R-GNB in the 90 days following the index visit. We compared patients with and without acquisition of R-GNB, using independent sample *t* tests or Wilcoxon rank-sum tests to compare continuous variables and χ^2^ tests to compare categorical variables. We also estimated the odds ratio of R-GNB acquisition for antibiotic exposure relative to no antibiotic exposure at the index visit. To adjust for additional risk factors, we used a multivariable logistic regression including patient characteristics, infectious diseases diagnoses, and recent healthcare utilization. All statistical analyses were performed using R version 4.0.1 software (R Foundation for Statistical Computing, Vienna, Austria) and implementing functions from the *epitab* package.^19^

## Results

### Patient characteristics

During the 2-year study period, 7,215 patients had qualifying visits at 13 outpatient clinic settings. Most patients were older adults (65.5 ± 14.2 years), male (90%), and White (84%). The most common comorbid condition was diabetes mellitus (35%), followed by chronic lung disease (25%). R-GNBs were detected in microbiological cultures of 206 (2.9%) of patients in the 7–90 days following their index visit (Table 1). These individuals, compared with those for whom no R-GNB were recovered, were slightly older (68.1 vs 65.4 years; *P* = .006), more likely to be female (17% vs 9%; *P* = .001), and more likely to be of Black race (19% vs 12%; *P* = .011). We did not detect significant differences in individual chronic conditions or in healthcare exposures prior to the index visit between those with and without R-GNBs detected.

### Outpatient visit characteristics

**Fig. 1.**
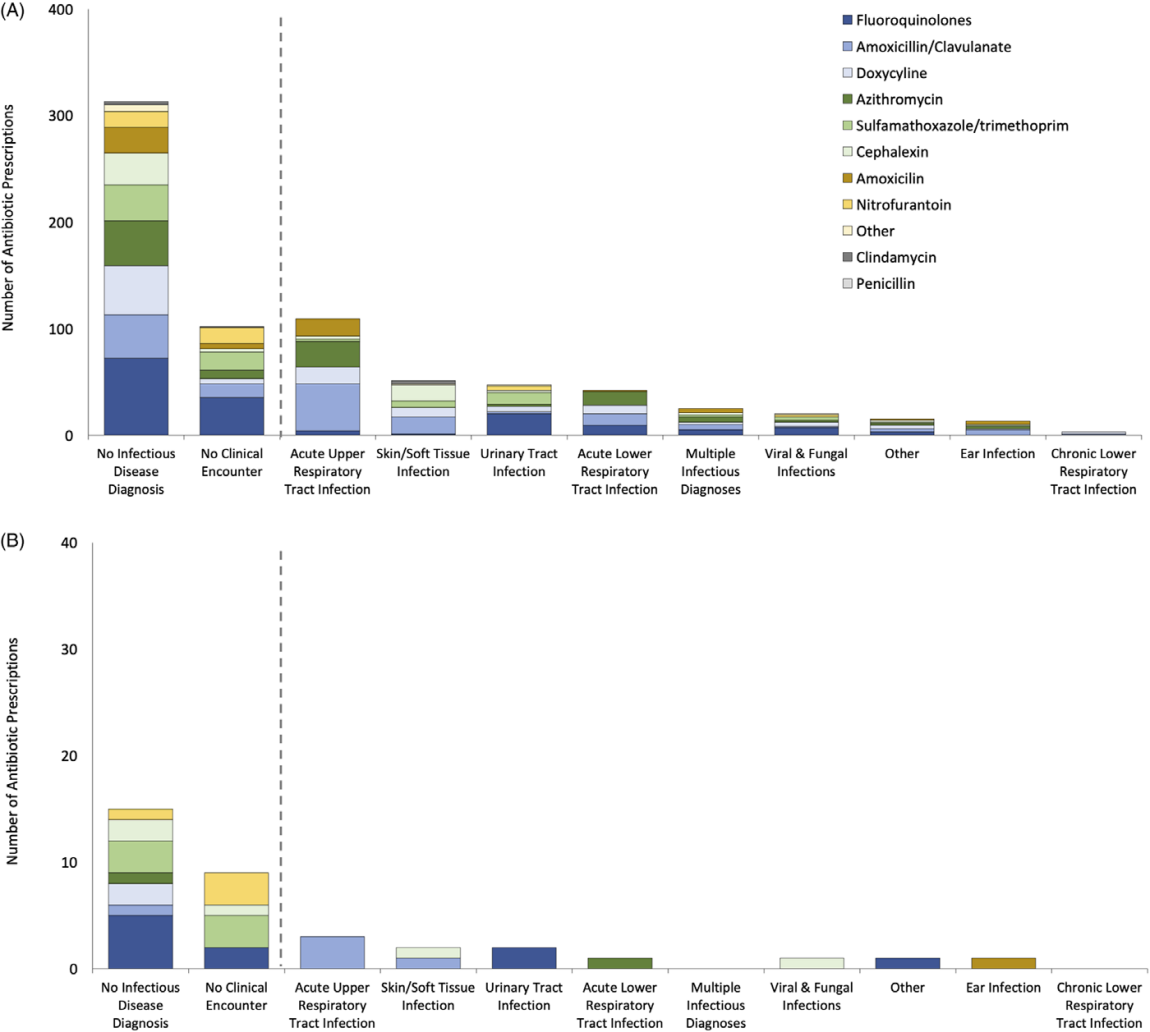
Diagnoses (according to *International Classification of Disease* (ICD) codes) and clinical encounters associated with antibiotic prescriptions issued to outpatients seen in community-based clinics associated with a large Veterans Affairs medical center in 2018–2019. (A) Antibiotic prescriptions that were not associated with a R-GNB. (B) Antibiotic prescriptions that were associated with an R-GNB. The list of antibiotics is ordered from most (fluoroquinolones) to least frequently prescribed (penicillin). Columns to the left of the vertical dashed line are for antibiotics prescribed at visits without an ICD code for an infection or antibiotics prescribed without documentation of a clinical encounter within the week before or following. Columns to the right of the dashed lines indicate infectious disease diagnoses based on ICD codes associated with the visit at which antibiotics were prescribed. Other includes the following: infections of the eye, heart and circulatory system, central nervous system, connective tissue, and bone and joint; neoplasms from infections; adverse effects of infectious disease treatments; post-operative infections; enteric infections; sexually transmitted infections; HIV; other bacterial infections; parasitic infections; and infections not otherwise specified.

Patients who acquired R-GNB were more likely to have been prescribed antibiotics at their index visit than those from whom no R-GNB were recovered (17% vs 10%; *P* = .004). Overall, the most commonly prescribed antibiotics were fluoroquinolones (Table 2). For all index visits associated with an antibiotic prescription, we assessed the diagnostic codes entered by the provider (Fig. 1). Notably, 57% of antibiotics prescribed either did not have an associated diagnostic code [328 (43%) of 768 prescriptions from 745 visits] or were issued outside of a clinical visit [>1 week removed from documentation of an in-person or telehealth visit; 111 (14%) of 768]. For antibiotics prescriptions accompanied by a diagnostic code, the most common indications overall were acute upper respiratory tract infections (15%), skin and soft-tissue infections (7%), urinary tract infections (7%), and acute lower respiratory tract infections (6%).

**Table 2.** Antibiotics Prescribed at Index Visits^a^

Antibiotic	No Subsequent Resistant Gram-Negative Bacteria (n = 711 visits), No. (%)	Subsequent Resistant Gram-Negative Bacteria (n = 34 visits), No. (%)
Fluoroquinolones	153 (22)	10 (29)
Amoxicillin–clavulanate	141 (20)	5 (15)
Doxycycline	101 (14)	2 (6)
Azithromycin	100 (14)	2 (6)
Sulfamethoxazole–trimethoprim	77 (11)	6 (18)
Cephalexin	57 (8)	5 (15)
Amoxicillin	54 (8)	1 (3)
Nitrofurantoin	34 (5)	4 (12)
Clindamycin	6 (1)	0 (0)
Penicillin	1 (0)	0 (0)
Other^b^	9 (1)	0 (0)

a The number of antibiotic prescriptions exceeds the number of visits because >1 antibiotic may have been issued at a single visit.

b Other includes ampicillin (n = 5), minocycline (n = 3), and clarithromycin (n = 1).

### Risk factors for acquisition of R-GNB

In our cohort, the majority of cultures from which R-GNB were isolated were from urine (66%) or a rectal swab (27%) collected prior to a prostate biopsy. Table 3 details the R-GNB recovered from clinical cultures, the most common of which were *E. coli* resistant to β-lactam–β-lactamase inhibitor combinations. Upon manual review of the chart of a subset of 30 patients, we found 100% agreement with the susceptibility patterns of R-GNB identified using clinical and administrative electronic records.

**Table 3.** Gram-Negative Bacteria Recovered from Clinical Cultures of Outpatients^a,b^

Phenotypes	*Escherichia coli* (n = 378), No. (%)	*Klebsiella* spp (n = 114), No. (%)	*Proteus* spp (n = 46), No. (%)	*Pseudomonas aeruginosa* ^c^ (n = 60), No. (%)	*Enterobacter* spp (n = 21), No. (%)	Other Gram-Negative Bacteria^d^ (n = 26), No. (%)
Wild type	216 (57)	82 (72)	31 (67)	52 (87)	20 (95)	24 (92)
Fluoroquinolones^c^	96 (25)	6 (5)	9 (20)	3 (5)	0 (0)	1 (4)
Extended-spectrum cephalosporins	18 (5)	6 (5)	2 (4)	1 (2)	1 (5)	0 (0)
β-lactam–β-lactamase inhibitor combinations	125 (33)	32 (28)	6 (13)	0 (0)	1 (5)	1 (4)
Carbapenems	0 (0)	0 (0)	2 (4)	6 (10)	0 (0)	0 (0)

a Some patients had multiple isolates from cultures that met our criteria following their index visit and some isolates were resistant to >1 class of agents and contribute to the counts in >1 row.

b Nonsusceptibility to individual agents indicated resistance to a class of antibiotics, as follows: ciprofloxacin, levofloxacin, or moxifloxacin for fluoroquinolones; ceftriaxone or cefepime or ESBL for extended-spectrum cephalosporins; ampicillin/sulbactam (only for *Escherichia coli*, *Klebsiella* spp, *Citrobacter koseri*, or *Proteus* spp) or piperacillin–tazobactam (for all GNB assessed) for β-lactam–β-lactamase inhibitor combinations; ertapenem, meropenem, or imipenem–cilastin for carbapenems.

c *P. aeruginosa* resistance to fluoroquinolones, extended-spectrum cephalosporins, BL–BLI combinations, and carbapenems was based only on the following agents, respectively: ciprofloxacin, cefepime, piperacillin–tazobactam, and imipenem–cilastin.

d Includes *Serratia marcescens* (n = 8), *Citrobacter* spp (n = 11), and *Providencia* spp (n = 7).

### Regression model

In an unadjusted logistic model predicting R-GNB with antibiotic exposure, the odds of acquiring a R-GNB in the 7–90 days following a primary care visit accompanied by any antibiotic prescription were significantly greater than the odds following primary care visits without an antibiotic prescription (unadjusted odds ratio [OR], 1.75; 95% confidence interval [CI], 1.18–2.52). Even after adjusting for other factors that were significantly associated with R-GNB acquisition (ie, female sex, increasing age, and Black race), the odds ratio for R-GNB acquisition following a primary care visit with an antibiotic prescription remained statistically significant (OR, 1.66; 95% CI, 1.11–2.41; *P* = .010) (Table 4).

**Table 4.** Results of Multivariable Logistic Regression Evaluating Risk Factors for Acquiring Resistant-Gram Negative Bacteria

Variable	Odds Ratio (95% CI)^a^	*P* Value
Sex, male vs female	0.381 (0.254–0.587)	**<.001**
Age, 1-y increments	1.026 (1.014–1.038)	**<.001**
**Race**		
White, reference	1.000	…
Black	1.876 (1.287–2.673)	**.001**
Other^a^	0.758 (0.301–1.626)	.515
**Ethnicity**		
Not Hispanic, reference	1.000	…
Hispanic	1.015 (0.244–2.812)	.980
Other^b^	1.439 (0.477–3.493)	.465
Charlson score (1-point increments)	1.003 (0.923–1.086)	.939
**Healthcare events in the year prior to the index visit**		
Any outpatient antibiotic prescription^c^	1.160 (0.801–1.648)	.417
Any hospital admission^c^	1.572 (0.876–2.681)	.111
Any emergency department visits	0.705 (0.430–1.109)	.147
High no. (>12) of specialty care visits	0.687 (0.439–1.048)	.091
High no. (>4) of primary care visits	0.758 (0.507–1.104)	.161
Antibiotic prescribed at index visit	1.661 (1.114–2.405)	**.010**

Note. CI, confidence interval.

a Other race includes American Indian, Alaska Native, Asian, Native Hawaiian or Pacific Islander and unknown.

b Other ethnicity includes unknown.

c Accounts for the year prior to the index visit; patients who were discharged or who received outpatient antibiotics ≤90 days prior to their index visit were excluded from analysis.

## Discussion

In this retrospective cohort study to assess the prevalence and risk factors related to outpatients with R-GNB, we detected a previously unreported R-GNB among 2.9% of outpatients. These patients had not had a recent hospital or nursing home admission and had not had an antibiotic prescription in the 90 days preceding a primary care visit. We also observed that although R-GNB acquisition only occurred in a small proportion of the cohort, the prescription of an antibiotic at the index visit increased the risk of acquiring a R-GNB by nearly 2-fold. Furthermore, more than half of those antibiotic prescriptions were issued without a coded infectious disease diagnosis or a clinical encounter recorded in the medical record. Taken together, the results of our study underscore the need for improved documentation of antibiotic indications and continued antibiotic stewardship efforts in outpatient settings to curtail the use of antibiotics that may be unnecessary and thereby prevent the acquisition of R-GNB.

The emergence of antibiotic resistance in the outpatient setting has been reported as soon as 1 month after antibiotic exposure, and it can occur as much as 1 year after the index antibiotic prescription.^20,21^ The emergence of antibiotic resistance in the outpatient setting is frequently associated with increased healthcare exposures, occurring within 90 days after inpatient admission and intravenous antibiotic treatment.^22,23^ In contrast, this study characterizes a cohort of outpatients who acquired R-GNB and neither received antibiotics nor had healthcare exposure in the previous 90 days. As observed in other studies that focus on patterns of antibiotic prescribing in the outpatient setting, >50% of patients in our cohort did not have an infectious disease diagnosis associated with their antibiotic prescriptions.^24,25^ Given that millions of outpatient antibiotic prescriptions are issued annually^26^ and that the impact of those prescriptions toward the emergence of antibiotic resistance can persist for months, the contribution of outpatient antibiotic exposure to the overall burden of antibiotic resistance is likely to be significant and grossly underestimated.

R-GNB recognized via clinical cultures may be difficult to associate with a specific healthcare episode or antibiotic exposure because many are bacterial species that are commensal organisms in the gut microbiota; thus, the duration of colonization may be prolonged Furthermore, horizontal transmission of R-GNB and resistance determinants can occur even in the absence of antibiotic selective pressure.^27,28^ In our cohort, most of the new R-GNB acquisitions were not associated with an antibiotic exposure, suggesting that microbiological cultures obtained after the index visit revealed pre-existing colonization among many individuals. Those patients were older, were more likely to be female, and were more likely to come from a racial minority than those without R-GNB detected.

*Escherichia coli* resistant to BLs and BLIs were the most common R-GNB in our cohort. This finding is particularly worrisome because amoxicillin–clavulanic acid, a representative of this class of drugs, is a key antibiotic in the armamentarium used in the outpatient setting, as our data demonstrate. The presence of ESBL-producing bacteria may also be important, which is consistent with several contemporary reports indicating an increase in this type of infections among patients hospitalized in the United States that is largely driven by community-associated infections.^5–8^ Resistance to fluoroquinolones was also fairly common, particularly among *E. coli* and *Proteus* spp, while carbapenem-resistance was rare and mostly a feature of *Pseudomonas aeruginosa* isolates. Notably, rectal screening cultures were routinely obtained to guide prophylaxis for invasive urologic procedures, most commonly prostate biopsies, which is a common procedure among older men. Cultures consistent with this practice accounted for ∼25% of R-GNB; in the absence of this practice, a lower rate of R-GNB may have been observed.

Due to both their association with the *C. difficile* infections and with risks for serious adverse events, meriting a “black box warning” by the US Food and Drug Administration, fluoroquinolones have been a particular target for antimicrobial stewardship efforts, yet they were the most common antibiotic prescribed to our cohort.^29–32^ Amoxicillin–clavulanate was also among the most common agents used, particularly for acute upper respiratory tract infections and for visits without a documented infectious disease diagnosis. This finding suggests that, similar to observations in hospitals, clinicians may prescribe antibiotics to reduce the cognitive load associated with diagnostic uncertainties in patients with suspected infection.^33^ As previously reported by Young et al,^24^ the large proportion of antibiotics prescribed without an accompanying ICD code has notable implications for implementing antimicrobial stewardship in outpatient settings. It is possible that prescribers may include the rationale for antibiotic use in their clinical notes. The inability to link these prescriptions with specific diagnoses, however, can hinder effective measurement of antibiotic use for certain indications, which in turn limits the opportunity to implement changes in antibiotic use. The proportion of antibiotics issued without a documented clinical interaction raises the possibility that patients may be initiating contact, reporting symptoms, and receiving antibiotics outside of the clinical and electronic medical record documentation structures. Similar to lack of associated ICD codes, prescribing antibiotics by telephone can also hinder effective antibiotic stewardship efforts.^34,35^ Simply stated, we cannot change what we cannot measure.

Our study has several limitations. First, the work was performed at a single VA healthcare system, which may limit the generalizability of the results. VA healthcare users differ from the general US population in that they are predominantly older, White, non-Latino males with a higher burden of chronic medical conditions.^36,37^ Furthermore, the population served by the VA NEOHS may differ from that of other VAMCs. Second, some veterans access both VA and non-VA healthcare services, meaning that our results may underestimate antibiotic exposures, hospitalizations, and R-GNB not captured in the VA administrative database. At the Indianapolis VA health system, estimates posit that <20% of veterans received prescriptions from non-VA sources; of those, 17.5% were for antibiotics. In 2014, the ratio of veterans aged ≥65 years who accessed primary care through the VA only compared to VA and Medicare services (dual users) was ∼4:1.^38^ Third, this retrospective cohort study relied on administrative data, which have inherent limitations, for example, lack of certainty regarding whether or not patients took the antibiotics prescribed. Finally, our approach only accounted for new R-GNB acquisitions among patients via clinical cultures, which may have underestimated the number of individuals colonized with these organisms.

Despite these limitations, the findings presented here shed new light on the risk factors associated with community-acquired R-GNB in the context of a comprehensive healthcare system. Although hospitalizations during the year prior contributed, antibiotics prescribed during the index outpatient visit also contributed. Curtailing unnecessary antibiotic exposure, which includes using narrow-spectrum agents as well as avoiding prescriptions altogether, is the most readily modifiable risk factor that clinicians can use to improve the health and safety of their patients. Our results also suggest 2 specific actions prescribers could take to help improve antibiotic stewardship in the outpatient setting. The first action is to associate diagnostic codes with the clinical encounter that led to an antibiotic prescription. The second action is to avoid prescribing these agents in the absence of a clinical encounter. This recommendation does not preclude the use of telehealth but does call for documentation of the evaluation and clinical decision-making process involved with the antibiotic prescribing event. Given that most healthcare visits take place in outpatient settings, improved efforts to reduce unnecessary antibiotic exposures in that sphere can yield a considerable reduction on the acquisition of R-GNB and potentially enhance the health of a large number of individuals.

## Supplementary material

For supplementary material accompanying this paper visit https://doi.org/10.1017/ash.2021.231. click here to view supplementary material
