# Supplementary material for: Antibiotic exposure and acquisition of antibiotic-resistant gram-negative bacteria among outpatients at a US Veterans Affairs medical center
Source: Antimicrob Steward Healthc Epidemiol. 2022 Jan 12;2(1):e5. doi: 10.1017/ash.2021.231 (PMC9615019; doi:10.1017/ash.2021.231)
Supplement: Supplementary file 1 [file S2732494X2100231Xsup001.docx]

Supplemental Tables 1A-E

| **Supplemental Table 1A. Antibiotic Susceptibilities of Common Gram-Negative Bacteria, wild-type** | | | | | | | | |
| --- | --- | --- | --- | --- | --- | --- | --- | --- |
| **Antibiotics** | Escherichia coli | Klebsiella spp. | Enterobacter spp. | Citrobacter koseri | Serratia spp. | Proteus spp. | Providencia spp. | Pseudomonas aeruginosa |
| ampicillin | S | - | - | - | - | S | - | - |
| cefazolin | S | S | - | S | - | S | - | - |
| ceftriaxone | S | S | S | S | S | S | S | - |
| cefepime | S | S | S | S | S | S | S | S |
| ciprofloxacin | S | S | S | S | S | S | S | S |
| ampicillin/sulbactam | S | S | - | S | - | S | - | - |
| piperacillin/tazobactam | S | S | S | S | S | S | S | S |
| ertapenem | S | S | S | S | S | S | S | - |
| imipenem | S | S | S | S | S | S | S | S |
| tetracycline | S | S | S | S | - | - | - | - |
| trimethoprim/sulfamethoxazole | S | S | S | S | S | S | S | - |
| nitrofurantoin | S | S | S | S | - | - | - | - |
|  |  |  |  |  |  |  |  |  |
| S, susceptible |  |  |  |  |  |  |  |  |
| -, wild-type not susceptible |  |  |  |  |  |  |  |  |
| R, resistant |  |  |  |  |  |  |  |  |

| **Supplemental Table 1B. Antibiotic Susceptibilities of Common Gram-Negative Bacteria, Fluoroquinolone-Resistance** | | | | | | | | |
| --- | --- | --- | --- | --- | --- | --- | --- | --- |
| **Antibiotics** | Escherichia coli | Klebsiella spp. | Enterobacter spp. | Citrobacter koseri | Serratia spp. | Proteus spp. | Providencia spp. | Pseudomonas aeruginosa |
| ampicillin | S | - | - | - | - | S | - | - |
| cefazolin | S | S | - | S | - | S | - | - |
| ceftriaxone | S | S | S | S | S | S | S | - |
| cefepime | S | S | S | S | S | S | S | S |
| ciprofloxacin | R | R | R | R | R | R | S | R |
| ampicillin/sulbactam | S | S | - | S | - | S | - | - |
| piperacillin/tazobactam | S | S | S | S | S | S | S | S |
| ertapenem | S | S | S | S | S | S | S | - |
| imipenem | S | S | S | S | S | S | S | S |
| tetracycline | S | S | S | S | - | - | - | - |
| trimethoprim/sulfamethoxazole | S | S | S | S | S | S | S | - |
| nitrofurantoin | S | S | S | S | - | - | - | - |
|  |  |  |  |  |  |  |  |  |
| S, susceptible |  |  |  |  |  |  |  |  |
| -, wild-type not susceptible |  |  |  |  |  |  |  |  |
| R, resistant |  |  |  |  |  |  |  |  |

| **Supplemental Table 1C. Antibiotic Susceptibilities of Common Gram-Negative Bacteria, Resistance to extended spectrum cephalosporins** | | | | | | | | |
| --- | --- | --- | --- | --- | --- | --- | --- | --- |
| **Antibiotics** | Escherichia coli | Klebsiella spp. | Enterobacter spp. | Citrobacter koseri | Serratia spp. | Proteus spp. | Providencia spp. | Pseudomonas aeruginosa |
| ampicillin | S | - | - | - | - | S | - | - |
| cefazolin | S | S | - | S | - | S | - | - |
| ceftriaxone | R | R | R | R | R | R | R | - |
| cefepime | R | R | R | R | R | R | R | R |
| ciprofloxacin | S | S | S | S | S | S | S | S |
| ampicillin/sulbactam | S | S | - | S | - | S | - | - |
| piperacillin/tazobactam | S | S | S | S | S | S | S | S |
| ertapenem | S | S | S | S | S | S | S | - |
| imipenem | S | S | S | S | S | S | S | S |
| tetracycline | S | S | S | S | - | - | - | - |
| trimethoprim/sulfamethoxazole | S | S | S | S | S | S | S | - |
| nitrofurantoin | S | S | S | S | - | - | - | - |
|  |  |  |  |  |  |  |  |  |
| S, susceptible |  |  |  |  |  |  |  |  |
| -, wild-type not susceptible |  |  |  |  |  |  |  |  |
| R, resistant |  |  |  |  |  |  |  |  |
|  |  |  |  |  |  |  |  |  |
| The mechanisms of resistance to extended-spetcrum cephalosporins (e.g., ceftriaxone, cefepime) vary among different bacteria. | | | | | | | | |

| **Supplemental Table 1D. Antibiotic Susceptibilities of Common Gram-Negative Bacteria, Resistance to beta-lactam/beta-lactamase inhibitor combinations** | | | | | | | | |
| --- | --- | --- | --- | --- | --- | --- | --- | --- |
| **Antibiotics** | Escherichia coli | Klebsiella spp. | Enterobacter spp. | Citrobacter koseri | Serratia spp. | Proteus spp. | Providencia spp. | Pseudomonas aeruginosa |
| ampicillin | S | - | - | - | - | S | - | - |
| cefazolin | S | S | - | S | - | S | - | - |
| ceftriaxone | S | S | S | S | S | S | S | - |
| cefepime | S | S | S | S | S | S | S | S |
| ciprofloxacin | S | S | S | S | S | S | S | S |
| ampicillin/sulbactam | R | R | - | R | - | R | - | - |
| piperacillin/tazobactam | R | R | R | R | R | R | R | R |
| ertapenem | S | S | S | S | S | S | S | - |
| imipenem | S | S | S | S | S | S | S | S |
| tetracycline | S | S | S | S | - | - | - | - |
| trimethoprim/sulfamethoxazole | S | S | S | S | S | S | S | - |
| nitrofurantoin | S | S | S | S | - | - | - | - |
|  |  |  |  |  |  |  |  |  |
| S, susceptible |  |  |  |  |  |  |  |  |
| -, wild-type not susceptible |  |  |  |  |  |  |  |  |
| R, resistant |  |  |  |  |  |  |  |  |
|  |  |  |  |  |  |  |  |  |
| The mechanisms of resistance to beta-lactam/beta-lactamase inhibitor combinations (e.g., ampicillin-sulbactam, piperacillin/tazobactam) vary among different bacteria. | | | | | | | | |

| **Supplemental Table 1E. Antibiotic Susceptibilities of Common Gram-Negative Bacteria, Carbapenem-Resistant Organisms** | | | | | | | | |
| --- | --- | --- | --- | --- | --- | --- | --- | --- |
| **Antibiotics** | Escherichia coli | Klebsiella spp. | Enterobacter spp. | Citrobacter koseri | Serratia spp. | Proteus spp. | Providencia spp. | Pseudomonas aeruginosa |
| ampicillin | S | - | - | - | - | S | - | - |
| cefazolin | S | S | - | S | - | S | - | - |
| ceftriaxone | S | S | S | S | S | S | S | - |
| cefepime | S | S | S | S | S | S | S | S |
| ciprofloxacin | S | S | S | S | S | S | S | S |
| ampicillin/sulbactam | S | S | - | S | - | S | - | - |
| piperacillin/tazobactam | S | S | S | S | S | S | S | S |
| ertapenem | R | R | R | R | R | R | R | - |
| imipenem | R | R | R | R | R | R | R | R |
| tetracycline | S | S | S | S | - | - | - | - |
| trimethoprim/sulfamethoxazole | S | S | S | S | S | S | S | - |
| nitrofurantoin | S | S | S | S | - | - | - | - |
|  |  |  |  |  |  |  |  |  |
| S, susceptible |  |  |  |  |  |  |  |  |
| -, wild-type not susceptible |  |  |  |  |  |  |  |  |
| R, resistant |  |  |  |  |  |  |  |  |
|  |  |  |  |  |  |  |  |  |
| For convenience, we grouped carbapenem-resistant organisms together. | | | | | | | | |
